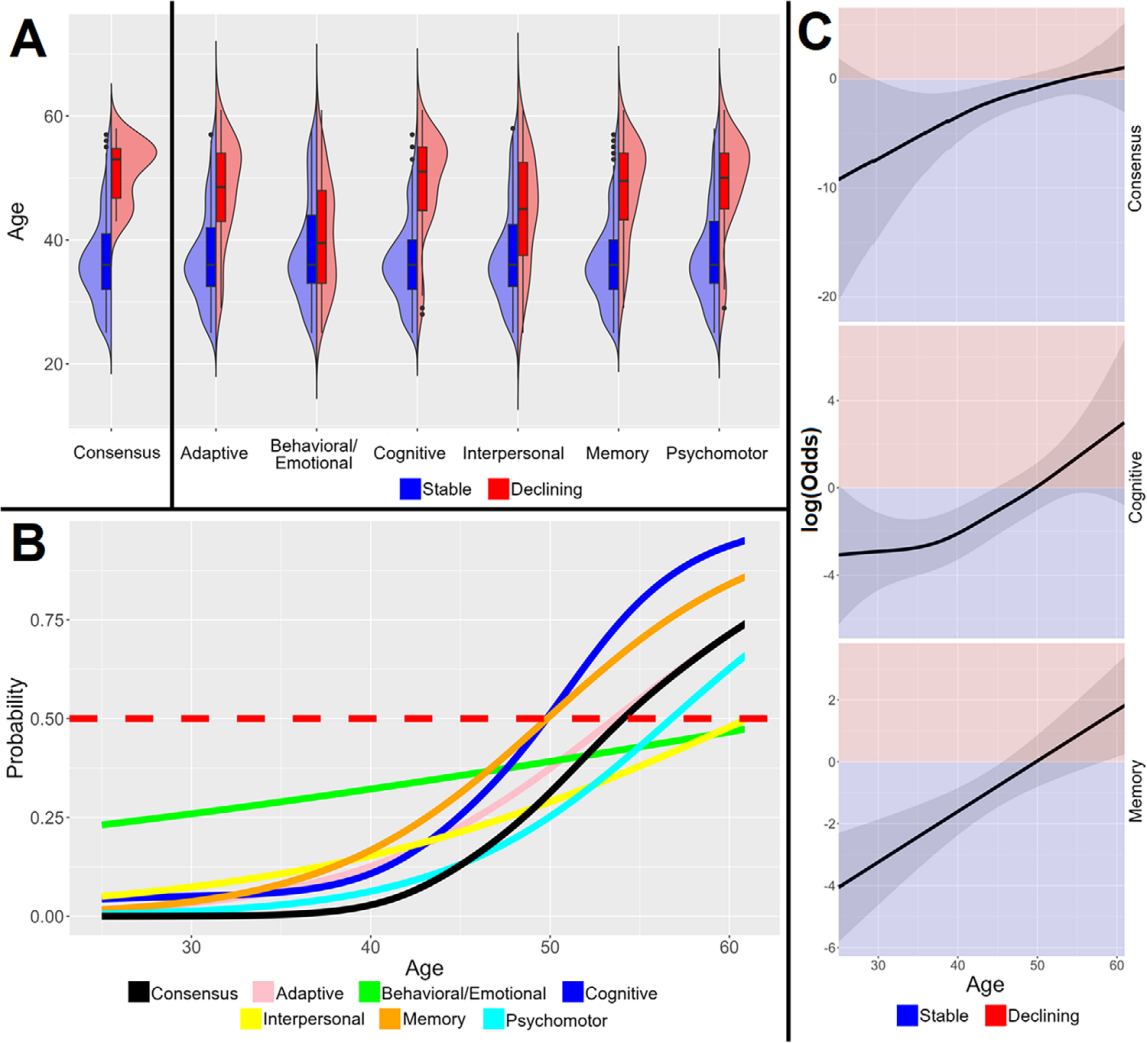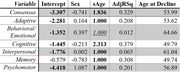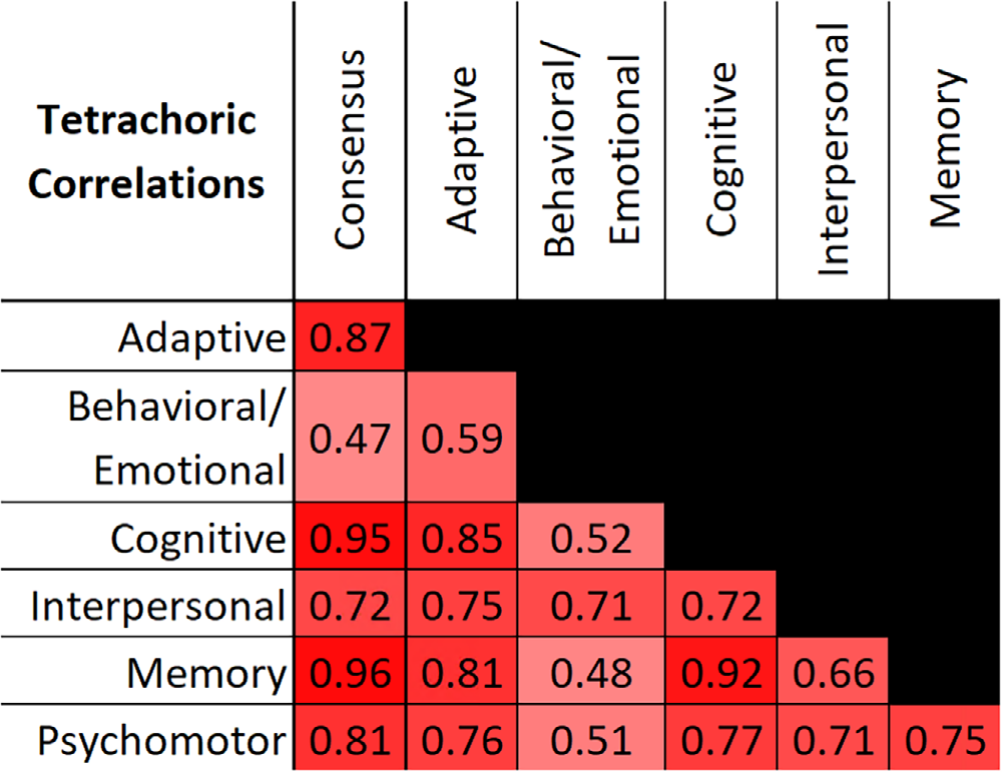# Domain Specific Age at Onset of Decline in Down Syndrome

**DOI:** 10.1002/alz.089191

**Published:** 2025-01-03

**Authors:** James Tyler Kennedy, Benjamin L Handen, Elizabeth Head, Mark Mapstone, Bradley T. Christian, Beau Ances

**Affiliations:** ^1^ Washington University in St. Louis School of Medicine, St. Louis, MO USA; ^2^ University of Pittsburgh, Pittsburgh, PA USA; ^3^ The UC Irvine Institute for Memory Impairments and Neurological Disorders (UCI MIND), Irvine, CA USA; ^4^ University of California, Irvine, Irvine, CA USA; ^5^ University of Wisconsin‐Madison, Madison, WI USA

## Abstract

**Background:**

Individuals with Down syndrome (DS) typically develop Alzheimer’s disease (AD) at an early age. Estimates of the age of decline vary, but typically place it in the early‐mid 50s. As AD onset can be difficult to identify in intellectually impaired cohorts, understanding the expected timing of decline may help individuals and caregivers prepare. It is unclear how timing in changes in different domains relates to the onset of AD dementia.

**Method:**

Data was obtained for individuals with DS (188 total, 53% male), ages 25 to 61, from the Alzheimer’s Biomarker Consortium–Down Syndrome and used in cross‐sectional analyses based on the baseline session. Decline was determined by performance on direct measures of cognition with participants and interviews of caregivers. Diagnosis of AD related decline was arrived at by group consensus. Domain‐specific decline was dichotomized as a stable vs decline binary for Adaptive, Behavioral/Emotional, Cognitive, Interpersonal, Memory, and Psychomotor changes. Consensus and domain‐specific data (Consensus n = 175, 18 declining, Domain n = 186‐187, 21‐60 declining) was available. The association between age and decline was analyzed in logistic generalized additive models (GAM), controlling for sex. Ages where individuals were equally likely to be stable or declining were extracted from GAM models. The relationship between domains/consensus was also examined using tetrachoric correlations. Results were considered significant at a p < .05 following multiple comparison correction.

**Result:**

Age was significantly associated with decline for all measures except the Behavioral/Emotional domain. AD related decline began at age 54, domain‐specific estimates varied: earliest in Memory (49.7) and Cognitive (49.8) and latest in Behavioral/Emotional (64.7). The relationship between age and decline probability was linear except for AD consensus where slope slows at age 44 and the Cognitive measure where slope increases at age 37. Age at decline did not differ between sexes. Correlations ranged from 0.96 (Memory with Consensus) to 0.47 (Consensus with Behavioral/Emotional).

**Conclusion:**

AD decline occurs at the age of 54 and is preceded by a change in cognition and memory ∼4 years earlier. Sex does not affect decline onset. Change in memory and cognition are closely related while behavioral/emotional changes are not strongly associated with AD‐related decline or other domains.